# Isolation, Bioactivity, and Production of *ortho*-Hydroxydaidzein and *ortho*-Hydroxygenistein

**DOI:** 10.3390/ijms15045699

**Published:** 2014-04-03

**Authors:** Te-Sheng Chang

**Affiliations:** Department of Biological Science and Technology, National University of Tainan, 33 Sec. 2 Su-Lin St., Tainan 702, Taiwan; E-Mail: mozyme2001@gmail.com; Tel./Fax: +886-6-260-2137

**Keywords:** soy isoflavones, daidzein, genistein, hydroxylation, *ortho*-hydroxydaidzein, *ortho*-hydroxygenistein, bioactivity, isolation, production, cancer, melanogenesis

## Abstract

Daidzein and genistein are two major components of soy isoflavones. They exist abundantly in plants and possess multiple bioactivities. In contrast, *ortho*-hydroxydaidzein (OHD) and *ortho*-hydroxygenistein (OHG), including 6-hydroxydaidzein (6-OHD), 8-hydroxydaidzein (8-OHD), 3′-hydroxydaidzein (3′-OHD), 6-hydroxygenistein (6-OHG), 8-hydroxygenistein (8-OHG), and 3′-hydroxygenistein (3′-OHG), are rarely found in plants. Instead, they are usually isolated from fermented soybean foods or microbial fermentation broth feeding with soybean meal. Accordingly, the bioactivity of OHD and OHG has been investigated less compared to that of soy isoflavones. Recently, OHD and OHG were produced by genetically engineering microorganisms through gene cloning of cytochrome P450 (CYP) enzyme systems. This success opens up bioactivity investigation and industrial applications of OHD and OHG in the future. This article reviews isolation of OHD and OHG from non-synthetic sources and production of the compounds by genetically modified microorganisms. Several bioactivities, such as anticancer and antimelanogenesis-related activities, of OHD and OHG, are also discussed.

## 1. Introduction

**Figure 1.**
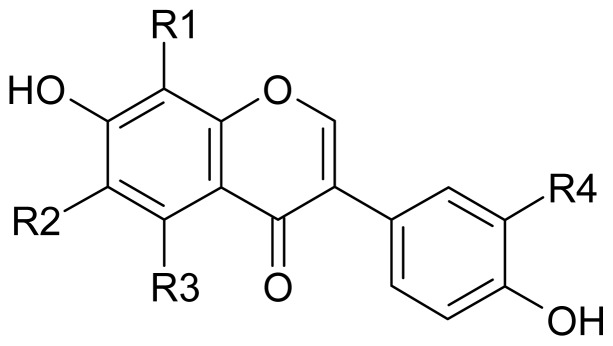
Structures of *ortho*-hydroxydaidzein (OHD) and *ortho*-hydroxygenistein (OHG). Daidzein, R_1_ = R_2_ = R_3_ = R_4_ = H; 6-Hydroxydaidzein (6-OHD), R_1_ = R_3_ = R_4_ = H, R_2_ = OH; 8-Hydroxydaidzein (8-OHD), R_1_ = OH, R_2_ = R_3_ = R_4_ = H; 3′-Hydroxydaidzein (3′-OHD), R_1_ = R_2_ = R_3_ = H, R_4_ = OH; Genistein, R_1_ = R_2_ = R_4_ = H, R_3_ = OH; 6-Hydroxygenistein (6-OHG), R_1_ = R_4_ = H, R_2_ = R_3_ = OH; 8-Hydroxygenistein (8-OHG), R_1_ = R_3_ = OH, R_2_ = R_4_ = H; 3′-Hydroxydaidzein (3′-OHG), R_1_ = R_2_ = H, R_3_ = R_4_ = OH.

Isoflavones are naturally occurring dietary phytoestrogens distributed in the leaves, seeds, bark, and flowers of some plants, specifically in legumes, such as soybeans [1]. In plants, these compounds provide protection against UV radiation, pathogens and herbivores [2]. Two major isoflavones found in soybeans are daidzin and genistin, which are the glycoside conjugates of daidzein and genistein, respectively. They account for more than 0.1% (*w*/*w*) of the dry weight of soybeans. In the past few decades, isoflavones have been intensively investigated due to their possible role in preventing certain hormone-dependent and other diseases including breast and prostate cancers, osteoporosis, and cardiovascular diseases [3]. In structure-activity relationships, the number and positions of functional groups in the chemical structures of isoflavones dramatically affect the functions of the isoflavones [4]. Therefore, many scientists focused on studies of isolation and bioactivity characterization of different derivatives of daidzein and genistein based on the multifunctional activities of soy isoflavones. Among various soy isoflavone derivatives, *ortho*-hydroxydaidzein (OHD) and *ortho*-hydroxygenistein (OHG) are an important group because of the *ortho*-dihydroxyl groups in their structures, which may enhance bioactivity compared with those of the precursors daidzein and genistein. Based on the difference in the hydroxylation position, OHD and OHG divide into 6-hydroxydaidzein (6-OHD), 8-hydroxydaidzein (8-OHD), 3′-hydroxydaidzein (3′-OHD), 6-hydroxygenistein (6-OHG), 8-hydroxygenistein (8-OHG), and 3′-hydroxygenistein (3′-OHG). The chemical structures of OHD and OHG together with daidzein and genistein are shown in Figure 1.

Although several thousands of different isoflavones have been purified and identified from plants, OHD and OHG have not been isolated from plants with the exception of 3′-OHD and 3′-OHG. Why hydroxylation at either the C-6 or C-8 carbon position of the isoflavone skeleton is not favored during isoflavone biosynthesis in plants is unknown. Most OHD and OHG are isolated from fermented soybean foods or microbial fermentation broth feeding with soybean meal. During fermentation, the microorganism possessing a unique cytochrome P450 enzyme system catalyzes *ortho*-hydroxylation of either daidzein or genistein into OHD and OHG, respectively. Different OHD and OHG can be isolated from different fermented soybean foods used by different microorganisms (which is discussed in detail below). To overcome the problem of the rarity of OHD and OHG in nature, in recent years, scientists successfully used genetically recombinant microorganisms harboring the cytochrome P450 (CYP) gene to produce OHD and OHG. This breakthrough will attract more scientists studying the bioactivity of OHD and OHG in the future because it is easier to produce OHD and OHG. Moreover, production of OHD and OHG by recombinant microorganisms can be easily scaled up, and OHD and OHG can be used for industrial applications. In this review, we surveyed reports on OHD and OHG from the past few decades. This review focuses on the isolation, bioactivity, and production of OHD and OHG, and is divided into those sections accordingly.

In addition, the absorption and metabolism of soy isoflavones daidzein and genistein by humans are also an important field of study. Many reports have demonstrated that OHD and OHG are intermediate metabolites of daidzein and genistein in humans and rats *in vitro* [5,6] or *in vivo* [7]. These reports have been thoroughly reviewed in other articles [8,9], and thus are not discussed in this review.

## 2. Isolation of OHD and OHG

The non-synthetic sources for isolating OHD and OHG can be divided into three groups: plants, fermented soybean foods, and microbial fermentation broth feeding with soybean meal. Only 3′-OHD and 3′-OHG have been found in plants, while other OHD and OHG were isolated from fermented soybean foods or microbial fermentation broth. In Asian countries, many traditional fermented products use soybeans as the fermented substrate, such as natto, soy sauces, and miso (Japanese fermented soybean paste), doenjang (Korean fermented soybean paste), douchi (Chinese fermented soybeans), and tempeh (Indonesian fermented soybean cake). Fungi are usually the major microorganisms involved in the preparation of these products. For examples, tempeh is produced mainly by *Rhizopus* sp. and others by *Aspergillus* sp. [10]. In addition, diverse bacteria are also involved in the preparation of Indonesian tempeh and Korean doenjang. Many studies have been conducted on the metabolism of soy isoflavones by the fungi in these fermented soybean foods [11,12]. The results showed that each isoflavone glycoside was hydrolyzed to the respective free isoflavone aglycone by fungal beta-glucosidase during soybean fermentation. Furthermore, during fermentation, free isoflavone daidzein and genistein are biotransformed into OHD and OHG, respectively, by enzymes from the microorganisms [13,14]. The catalyzing enzyme has been identified as CYPs [15,16]. CYPs are heme-containing monooxygenases wildly distributed in nature, including microorganisms, plants, and animals [17]. CYPs from humans [18,19], murine [20], and microorganisms [16,21] can biotransform daidzein or genistein into OHD and OHG, respectively. However, CYPs from plants cannot catalyze *ortho*-hydroxylation of daidzein or genistein. The phenomenon reasonably explains the fact that rare OHD and OHG were isolated from plants; nevertheless, the detailed mechanism is unknown. In addition to fermented soybean products, OHD and OHG can also be purified from the fermentation broth of microorganisms containing CYPs to catalyze *ortho*-hydroxylation of daidzein or genistein, when soybean meal is used as a substrate. Furthermore, OHD and OHG were recently produced by using genetically modified microorganisms harboring CYPs through the biotransformation of daidzein or genistein. This will be discussed later in this review. The isolation of OHD and OHG from three non-synthetic sources is shown in Table 1.

Among various OHD and OHG, 3′-OHG (orobol) from *Orobus tuberuosus* was the first to be purified in nature, in 1939 [22]. Then, 3′-OHG was isolated from several other plants, including the seeds of *Calophyllum polyanthum* [23], the stems of *Erycibe expansa* [24], whole plants of *Eclipta prostrate* [25], and the flowers of *Sophora japonica* (SophoraeFlos) [26]. In addition to plants, 3′-OHG was also isolated in tempeh [27] and the fermentation broth of *Aspergillus niger* [28]. In contrast, 3′-OHD was initially isolated from the heartwood of *Machaerium villosum* in 1968 [29] and then from the heartwood of *Dalbergia odorifera* [30] and identified as the single major flavonoid in fruits of *Styphnolobium japonicum* [31]. In addition, 3′-OHD exists in most fermented soybean products.

6-OHD, until now isolated only from fermented soybeans, was purified from tempeh for the first time in 1964 [32] and then isolated from every fermented soybean product, including Japanese soybean koji [33,34], Japanese miso [35–38], Chinese douchi [39], and Korean doenjang [40]. For fermented soybean foods prepared by using the fungal *Aspergillus* species, such as miso and douchi, OHD and OHG were produced by CYP57B3 from the microorganism [15]. However, for tempeh, OHD and OHG are not produced by the fungal *Rhizopus* sp. in the tempeh but by bacteria isolated from tempeh [41,42]. The enzyme of the bacteria for the biotransformation of OHD and OHG from daidzein and genistein remains unknown. In addition, the microorganism in Korean doenjang that produces OHD has not been identified.

8-OHD and 8-OHG were initially isolated from microbial fermentation broth: 8-OHG from the cultivation of *A. niger* in 1975 [28] and 8-OHD from the cultivation of *Streptomyces* sp. in 1989 [43,44]. Similar to 3′-OHD and 6-OHD, 8-OHD and 8-OHG have been isolated from almost every fermented soybean product.

Among all OHD and OHG, 6-OHG is rarely discovered in non-synthetic sources. Klus and Barz identified 6-OHG as the metabolite of genistein with tempeh-derived bacterial *Micrococcus* or *Arthrobacter* in 1998 [45]. However, the catalyzing enzyme in the bacteria that produces 6-OHG has not been identified. Until now, there has been no report of production of 6-OHG by genetically modified microorganisms harboring CYPs. It seems that natural CYPs do not favor catalyzing 6-hydroxylation of genistein. The mechanism must be studied in the future. For the non-monooxylation catalyzing reaction, however, Tsuchihashi *et al.* isolated 6-OHG from the metabolites of human intestinal bacterial *Peptostreptococcus productus* by feeding the main isoflavone tectoridin (4′,5-dihydro-6-methoxy-7-(*o*-glucoside)isoflavone) from the flowers of *Pueraria thomsonii* [46]. The microbial enzyme from the *P. productus* strain caused *O*-demethylation at the C-6 methoxy group accompanied by hydrolysis of the glycosidic linkage substituted at the C-7 hydroxyl group. However, the production of 6-OHG from tectoridin by the *P. productus* strain is rarely applied in biotechnological use due to the difficulty growing the bacteria and rarity of the precursor in nature. Currently, the quantitatively pronounced conversions of genistein yielding 6-OHG by the two strains from tempeh are recommended for biotechnological production of the difficult-to-synthesize polyhydroxylated isoflavones.

## 3. Bioactivity of OHD and OHG

OHD and OHG possess excellent antioxidant and free radical-scavenging activities due to the *ortho*-dihydroxyl groups in their structures. The structure-activity relationships for antioxidant activity have been thoroughly discussed elsewhere [47]. In this section, other bioactivities, such as anticancer and antimelanogenesis-related activities, of OHD and OHG are listed in Table 2 and discussed below.

### 3.1. Anticancer-Related Bioactivity

Numerous *in vitro* and *in vivo* studies have shown that soy isoflavones exhibit antiproliferative activity against malignancies of the breast, colon, skin, and prostate [48–51]. In particular, genistein has received attention as a potential anticarcinogenic compound. Although it is generally thought that genistein and daidzein might play an important role in preventing these types of cancers, OHD and OHG might inhibit cancer growth rather than genistein and daidzein. For example, Spencer *et al.* found that genistein was selectively taken up into T47D tumorigenic breast epithelial cells and was subject to metabolism by CYP enzymes leading to the formation of 3′-OHG, which induced G2-M cell cycle arrest in T47D cells [52]. The authors found that the antiproliferative actions of 3′-OHG may be mediated by initial oxidative DNA damage, activation of ataxia telangiectasia and Rad3-related kinase (ATR), and downstream regulation of the p53 pathway leading to cell cycle arrest in G2-M [53]. The authors suggested this was one mechanism by which genistein exerts its anticancer activity *in vivo*.

Some literature reported the anticancer-related bioactivity of OHD and OHG together with the isolation of OHD and OHG from fermented soybean foods or fermentation broth. When Funayama *et al.* first isolated 8-OHD from the fermentation broth of *Streptomyces* sp., they reported that 8-OHD increased the life span of S180 mouse sarcoma-bearing mice [43]. Hirota *et al.* isolated various OHD and OHG from miso, and found that 8-OHG showed the most potent antimutagenic activity [36] and antiproliferative activity toward human promyelocytic leukemia cells [35].

Recently, Dong *et al.* reported several study results showing the anticancer-related activity of 6-OHD and 3′-OHD. They demonstrated that 6-OHD suppressed anchorage-dependent and -independent growth and induced cell cycle arrest at the S and G2/M phases in HCT-116 human colon cancer cells [54]. In a xenograft mouse model, the authors found that 6-OHD significantly decreased tumor growth and the volume and weight of HCT-116 xenografts by suppressing cyclin-dependent kinase 1 and 2 (CDK1 and CDK2) activity in tumors. The results suggest that CDK1 and CDK2 are potential molecular targets of 6-OHD to suppress HCT-116 cell proliferation *in vitro* and *in vivo*. For 3′-OHD, the authors showed that the molecule triggered cell cycle arrest at the G1 phase through attenuating the expression of CDK1 and displayed an antiproliferative effect against epidermal growth factor (EGF) receptor-positive skin cancer [55]. 3′-OHD also significantly inhibited UVB-induced cyclooxygenase 2 (COX-2) expression by suppressing NF-κB transcription activity in mouse skin epidermal JB6 P+ cells [56]. Furthermore, topical application of 3′-OHD clearly suppressed the incidence and multiplicity of UVB-induced tumors in hairless mouse skin. Taken together, these results provide insight into the anticancer activity of 3′-OHD as a potential skin cancer chemopreventive agent.

The development of multidrug resistance (MDR) to conventional chemoradiation therapy usually leads to failure in treating cancer. Accordingly, suppressing MDR takes advantage of decreased chemotherapy dosage and adverse effects, as well as improved efficacy in cancer therapy. Lo *et al.* demonstrated that 3′-OHD causes cell death in human cervical cancer cells through the ROS-dependent suppression of MDR transporters [57]. This effect of 3′-OHD significantly increased the cytotoxicity of epirubicin, an anticancer drug in human cervical cancer HeLa cells. In another study, researchers showed that 8-OHD effectively circumvents MDR in human colon adenocarcinoma Caco-2 cells through the ROS-dependent inhibition of efflux transporters [58]. These findings, together with those described above, revealed multiple functions of OHD and OHG in anticancer activity and high potency for development as cancer chemopreventive agents.

### 3.2. Antimelanogenesis-Related Bioactivity

Melanogenesis is a biosynthetic pathway for the formation of the pigment melanin in human skin. A key enzyme, tyrosinase, catalyzes the first and only rate-limiting steps in melanogenesis, and the down-regulation of enzyme activity guarantees the inhibition of melanogenesis. Because of the cosmetically important issue of hyperpigmentation, there is a big demand for tyrosinase inhibitors. This encourages researchers to seek potent tyrosinase inhibitors for cosmetic use [59].

We isolated several isoflavone derivatives from soygerm koji fermented with *A. oryzae* and demonstrated 6-OHD [60], 8-OHD, and 8-OHG [61] could be potent tyrosinase inhibitors. Among them, 6-OHD acted competitively 6-fold more than kojic acid on the l-tyrosine binding site of the enzyme while 8-OHD and 8-OHG irreversibly inactivated the enzyme and belong to the suicide substrates of tyrosinase with low partition ratios, low Michaelis constants, and high maximal inactivation rate constants [62]. To our knowledge, 8-OHD and 8-OHG are currently the most potent suicide substrates of mushroom tyrosinase and have high potential in application as a skin-whitening agent. 8-OHD was creamed in the appropriate condition and showed higher depigmenting activity than that of ascorbic acid 2-glucoside (AA2G) in an *in vivo* assay conducted with 45 volunteers by our laboratory [63].

In addition to our findings, Korean scientists have also conducted successful experiments in this field. Park *et al.* isolated several OHDs from Korean fermented soybean product [40], and confirmed the antimelanogenesis activity of 8-OHD and 3′-OHD [64]. Na *et al.* evaluated antimelanogenesis activity by using reconstituted human skin equivalents and demonstrated that the two OHDs can reduce pigmentation in African-American human skin equivalents more effectively than the standard skin-whitening drug, kojic acid [65]. In the report, they observed that antimelanogenesis activity of 8-OHD and 3′-OHD was exhibited through repression of microphthalmia-association transcription factor (MITF), the primary regulator for the expression of melanogenesis-related proteins [66]. Recently, Kim *et al.* identified 3′-OHD as a potent autophage inducer and confirmed that melanogenesis inhibition of 3′-OHD mediated activation of melanosome autophagy in melanocytes [67]. These study results together with our previous findings emphasize the application of 8-OHD and 3′-OHD in skin-whitening cosmetics; thus, mass production of OHD for industrial use should be established in the future. This issue will be discussed later in this review.

### 3.3. Other Bioactivity

In addition to anticancer and antimelanogenesis-related activities, reports on OHD and OHG with other bioactivities are also collected in Table 2. Among them, some assaying OHD and OHG were isolated from the three types of non-synthetic sources in Table 1. Tewtrakul *et al.* isolated 3′-OHG (orobol) from *E. prostrate* and identified potent HIV-1 integrase inhibitory activity, which supports the use of *E. prostrate* in AIDS treatment [25]. They also found that the compound down-regulated iNOS and COX-2 mRNA expression, which supports the traditional use of the plant for treating inflammatory-related disease [68]. Matsuda *et al.* isolated 3′-OHG from *E. expansa* and identified its hepatoprotective activity [24]. Tsuchihashi *et al.* also found that 6-OHG from the metabolite of human intestinal bacterial strain feeding with isoflavone tectoridin from *Pueraria* flowers has extremely potent hepatoprotective activity [46]. 8-OHD from Japanese soybean products was identified as an aldose reductase inhibitor, which is an attractive pharmacological target for treating diabetic complications [69].

Some studies focused on the bioactivity of 6-OHD and 3′-OHD, which were from organic synthesis. Recently, Seo *et al.* reported that 6-OHD at high concentrations (40 to 80 μM) suppressed adipogenesis in 3T3-L1 preadipocytes by directing targeting phosphatidylinositol 3-kinase (PI3K) [70]. In contrast, Chen *et al.* recently reported that 6-OHD at low concentrations (10 to 20 μM) significantly promoted 3T3-L1 preadipocytes differentiation [71]. The potential application of 6-OHD in either obesity or diabetes remains unclear. In the future, an *in vivo* study should be evaluated to clarify the effect of 6-OHD on adipocytes. In addition, Tasdemir *et al.* demonstrated that 6-OHD showed strong inhibition of the protozoa *Trypanosoma brucei rhodesiense* and *Trypanosoma brucei gambiense*, which cause human African trypanosomiasis, also known as sleeping sickness, and severe mortality and morbidity in sub-Saharan Africa [72]. In the same report, 3′-OHD also showed comparable antitrypanosomal activity. This finding was consistent with that by Salem and Werbovetz, who demonstrated that 3′-OHD displayed potent and selective toxicity against *Trypanosoma brucei brucei* [73]. In another study on atopic dermatitis (AD), a chronically relapsing skin disorder presenting with severe itching and inflammation, 3′-OHD improved AD symptoms, including visual clinical features, and reduced ear thickening and scratching behavior *in vivo*. Furthermore, orally administered 3′-OHD in the mouse model significantly inhibited epidermal thickening and infiltration of mast cells. The improved statuses were similar to those in the untreated control group [74]. In addition to this OHD and OHG bioactivity, more thorough research on the bioactivity of OHD and OHG will be reported in the future along with easier and increased production of OHD and OHG by genetically modified microorganisms.

## 4. Production of OHD and OHG

**Figure 2.**
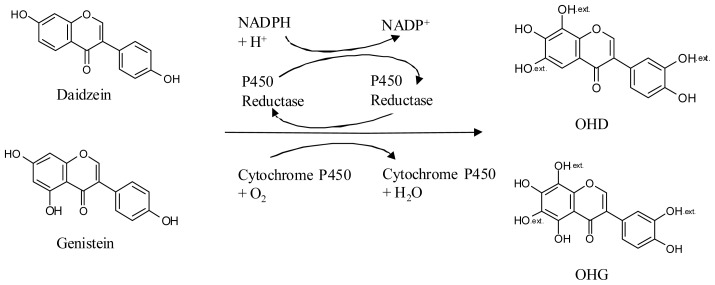
Production of OHD and OHG by the cytochrome P450 enzyme system. The functional groups (OH_.ext._) in the structures of the reaction products indicate possible hydroxylation positions.

In contrast to daidzein and genistein, which exist abundantly in plants, especially in soybeans, most OHD and OHG are biotransformed products from daidzein and genistein by microorganisms, where the CYP enzyme is the key enzyme. The biotransformation process is shown in Figure 2, where an electron transport chain is established from NADPH, P450 reductase, CYP, finally to a substrate. The *ortho*-hydroxylation of the precursors by CYP might occur at the C-6, C-8, or C-3′ position based on the regioselectivity of the enzyme.

Recently, some CYPs with the capacity to catalyze the *ortho*-hydroxylation of daidzein and genistein were identified. Many excellent studies have been performed by Kim *et al*. [21,75–84]. They subcloned every CYP in the genome of *Streptomyces avermitilis* MA-4680, *Nocardia farcinica* IFM10152, *Bacillus subtilis* 168, *B. megaterium*, *B. cereus*, and *B. licheniformis* and co-expressed the CYP with redox partners camA and camB from *Pseudomonas putida* in *Escherichia coli*. Then, the researchers screened the recombinant cells for the ability to catalyze *ortho*-hydroxylation of flavonoids [21]. Dozens of positive CYPs were identified; CYPs with the capacity to catalyze the *ortho*-hydroxylation of daidzein and genistein are listed in Table 3. CYP107H1 from *B. subtilis* catalyzed 3′-hydroxylation of daidzein; however, the production yield was very low [75]. The nfa33880 gene encoding CYP from *N. farcinica* catalyzed 6- and 8-hydroxylation of daidzein with low production yields [76]. Another CYP from the same microorganism (CYP154) showed distinct *O*-dealkylation and subsequent hydroxylation of formononetin (7-hydroxy-4′-methoxyisoflavone) to produce 3′-OHD [77]. Although the production yield was still low, this type of serial reaction mechanism is unusual among bacterial CYPs. In addition to the positive-screened CYP, Kim *et al.* also altered the substrate specificity of non-OHD-producing CYP (CYP102D1) from a long chain fatty acid to an aromatic compound such as daidzein through site-directed mutation on the active site of the enzyme. The mutating CYP102D1 (F96V/M246I) catalyzed 6- and 8-hydroxylation of daidzein [78]. The resulting production yield of 8-OHD (2.42 mg/L) in the study is the highest value in the literature.

Due to the low production yields of OHD and OHG, Kim *et al.* used two strategies to improve the yields [79,80]. First, the researchers changed the host to *Steptomyces*. Second, they genetically fused the CYP gene with the redox partner gene. During the catalysis process, CYP must be associated with electron donor partner proteins (CPRs) to transfer two electrons from NADPH to the CYP heme domain. However, the electron transfer between CPR and CYP usually limits the reaction rate of CYPs. One self-sufficient type of CYP is encoded in a single polypeptide that possesses a CPR domain fused to the heme domain [85]. Self-sufficient CYPs usually exhibit catalytic activity due to the high efficiency of the electron transfer from CPR to its fused P450 partner. Thus, genetic fusion of non-self-sufficient P450 with CPR mimics the natural self-sufficient CYPs and improves catalytic activity [86]. After performing the two strategies, Kim *et al.* improved 3′-OHD production by CYP105D7 from *S. avermitilis* higher than that of the wild-type strain and reached a production yield of 9.3 mg/L by using whole cell biotransformation [79] and 37.5 mg/L by using fermentation in a 7-L jar fermentor [80]. In addition to OHD, the only CYP discovered by Kim *et al.* that catalyzes *ortho*-hydroxylation of genistein was CYP107Y1 from *S. avermitilis* with a 3′-OHG production yield of 6.75 mg/L [21]. No study on the production of 8-OHG or 6-OHG by recombinant microorganisms has been reported. Screening new CYP from other microorganisms or genetically modifying CYP to alter its regioselection are two possible approaches for producing 8-OHG and 6-OHG in the future. In either approach, the high throughput screening (HTS) assay method is essential to obtain good results. The Kim research group recently developed a solid agar plate-based HTS assay method for screening *ortho*-specific hydroxylation of daidzein by sensing formaldehyde generated from the *O*-dealkylation reaction. Through the technique, they successfully screened one mutant G1 (A273H/G274E/T277G) from CYP102D1 with a 4-fold increase production yield of 3′-OHD (14.3 mg/L) compared with that of CYP102D1 (F96V/M246I) [81]. The fascinating technique would be an ideal system for primary HTS assay for the CYP reaction.

In addition to heme-containing monooxygenase (CYP), the Kim research group recently used recombinant *E. coli* harboring a non-heme, flavin-dependent monooxygenase (Sam5) from *Saccharothrix espanaensis* to produce 3′-OHD with a production yield of 75 mg/L [82]. The recombinant strain catalyzed multiple flavonoids to produce corresponding *ortho*-hydroxyflavonoids, including 3′-OHG, 3′,8-dihydroxygenistein, 3′-hydroxyapigenin, 3′,8-dihydroxyapigenin and 3′-hydroxynaringenin. The regioselective hydroxylation of diverse flavonoids shown by the recombinant strain has not been shown by other bacterial monooxygenase. Hence, the Sam5 system has highly potency for production of bioactive hydroxylated flavonoids. Because the production yield of OHD by the Sam5 system was greatly higher than those by bacterial CYP system, this finding opens further research on non-heme monooxygenase for production of OHD and OHG in the future.

As described in Section 2 of this review, *A. oryzae* is the most commonly used microorganism in fermented soybean products. The microorganism transforms soy isoflavones into their corresponding *ortho*-hydroxyl derivatives. *A. oryzae* contains 155 putative P450 genes in its genome, from which 142 P450 proteins are expressed [15]. Among them, CYP57B3 was recently shown to catalyze 3′-hydroxylation of genistein in cooperation with a CPR from *Saccharomyces cerevisiae* [16]. However, the authors did not measure the production yield of 3′-OHG in their study. To improve production, we recently fused CYP57B3 with the reductase domain of a self-sufficient CYP102A1 gene from *B. megaterium* to form an artificial, self-sufficient P450. Through measuring the production of *ortho*-hydroxydaidzein derivatives from daidzein with recombinant *P. pastoris* harboring the fusion gene, 6-OHD was produced as high as 9.1 mg/L, the highest production yield in the literature [87]. In addition, recombinant *P. pastoris* also produced a large amount of 3′-OHG [88].

CYP57B3 is the only CYP from eukaryotic cells that can produce OHD and OHG; others are from bacteria. Using the eukaryotic CYP expressed in yeast to produce OHD or OHG has several advantages compared to using bacterial CYP expressed in *E. coli* or *Streptomyces*. First, all bacterial CYPs are soluble and distributed in cytoplasm, while eukaryotic CYPs are membrane-anchored and mainly distributed on the endoplasmic reticulum membrane. For the insoluble substrate daidzein and genistein, which are transported via the endomembrane system in cells, membrane-anchored CYPs can access the substrate more easily than soluble bacterial CYPs. In fact, the production yields of OHD and OHG using bacterial CYP expressed in *E. coli* are usually very low [75–78]; Second, to improve the production of OHD and OHG in the bacterial system, the host could be changed to *Streptomyces* strains, which produce moderate amounts of OHD and OHG and are more suitable hosts than *E. coli*. However, *Streptomyces* contains not only CYP catalyzing *ortho*-hydroxylation of daidzein and genistein but also several dioxygenases that break down the precursors daidzein and genistein and the products OHD and OHG. Thus, OHD and OHG products in recombinant *Streptomyces* are not stable [83]. In contrast, the precursors and products are stable in yeast [89]. Although the enzyme catalyzing degradations of hydroxyisoflavones in *Streptomyces* was recently identified as tyrosinase, however, *ortho*-hydroxylation of daidzein in a tyrosinase gene-knock out mutant (Δ*melC2*) appeared to show little daidzein hydroxylation activity due to a low expression level of the CYP gene [84]. The reason may be related to the function of the tyrosinase with sporulation and cell stress environment. The drawback of using *Streptomyces* as a host to produce OHD and OHG needs to be resolved in the future; Third, CYP57B3 has been shown to catalyze the conversion of the isoflavone genistein, the flavanone naringenine [16], and the isoflavone daidzein [87]. However, the CYPs from bacteria catalyzed only one type of flavonoid as a substrate for each CYP [21]. These results reveal that CYP57B3 has greater flexibility in substrates binding than those of the CYPs from bacteria. The board substrate spectrum of the eukaryotic CYP57B3 provides CYP more application opportunities.

## 5. Conclusions

Small amounts of OHD and OHG are usually isolated from natural sources, if they exist. The rare isolation of OHD and OHG has limited the investigation of the bioactivities of the compounds. The collective reports describing bioactivity in this review show that more studies evaluating the bioactivity of OHD and OHG were completed in the last five years (2009–2013) compared with the previous decade. The trend should continue because of the easier availability of OHD and OHG from microbial production via genetic engineering. Daidzein and genistein are inexpensive and available in kilogram-grade with 99% purity. After OHD and OHG are produced from daidzein and genistein by recombinant microorganisms harboring necessary CYPs, industrial-scale production can be achieved by scaling up the fermentation process. Moreover, bioconversion of daidzein and genistein into OHD and OHG, respectively, by genetically modified microorganisms could be improved through multiple approaches, such as searching for new CYPs with higher catalyzing activity, improving the catalyzing capacity of CYPs by various mutation and HTS methods, and optimizing the fermentation control on a larger scale. Overall, more bioactivity evaluations and larger-scale production promise real applications of OHD and OHG in the future.

## Figures and Tables

**Table 1.** Isolation of OHD and OHG. Products that are underlined were isolated from non-synthetic sources for the first time.

Sources	Name	Microorganisms	Products	Ref.
**Plants**	*Orobus tuberuosus*	--- a	3′-OHG	[22]
	*Calophyllum polyanthum*	---	3′-OHG	[23]
	*Erycibe expansa*	---	3′-OHG	[24]
	*Eclipta prostrata*	---	3′-OHG	[25]
	*Sophora japonica*	---	3′-OHG	[26]
	*Machaerium villosum*	---	3′-OHD	[29]
	*Dalbergia odorifera*	---	3′-OHD	[30]
	*Styphnolobium japponicum*	---	3′-OHD	[31]

**Soybean Foods**	Indonesian Tempeh	*Rhizopus* and other bacteria	6-OHD, 8-OHD, 3′-OHG	[27,32]
	Japanese Soybean koji	*A. saitoi*	8-OHD, 8-OHG, 6-OHD	[33,34]
	Japanese Miso	*A. oryzae*	8-OHD, 8-OHG, 6-OHD, 3′-OHD	[35–38]
	Chinese Douchi	*A. oryzae*	6-OHD, 8-OHD, 3′-OHD, 8-OHG	[39]
	Korean Doenjang	diverse fungi and bacteria	6-OHD, 8-OHD, 3′-OHD	[40]

**Microbial Fermentation Broth**	---	*A. niger*	8-OHG, 3′-OHG	[28]
	---	*Streptomyces* sp.	8-OHD, 3′-OHD	[43,44]
	---	*Micrococcus* sp. or *Arthrobacter* sp.	6-OHG	[45]
	---	*P. productus*	6-OHG	[46]

a not involved.

**Table 2.** Bioactivity of OHD and OHG.

Classification	Compound	Bioactivity	Ref.
**Anticancer-Related Activity**	3′-OHG	Antiproliferative activity toward T47D tumorigenic breast epithelial cells;	[52,53]

	8-OHD	Increases life span against S180 bearing mice;	[43]
		Suppression of MDR in Caco-2 colon adenocarcinoma cells;	[58]

	8-OHG	Antimutagenesis activity;	[36]
		Antiproliferative activity toward HL-60 promyelocytic leukemia cells;	[35]

	6-OHD	Suppression of HCT-116 colon cancer cell proliferation *in vitro* and *in vivo*;	[54]

	3′-OHD	Suppression of EGF receptor-positive skin cancer cell proliferation *in vitro* and *in vivo*;	[55,56]
		Suppression of MDR transporters;	[57]

**Antimelanogenesis- Related Activity**	6-OHD	Competitive tyrosinase inhibitor;	[60]

	8-OHD	Irreversible tyrosinase inhibitor;	[61,62]
		Antimelanogenesis activity in human volunteers;	[63]

	8-OHG	Irreversible tyrosinase inhibitor;	[61,62]

	3′-OHD	Antimelanogenesis activity in human skin equivalents;	[64,65]
		Potent autophage inducer;	[67]

**Other Bioactivities**	3′-OHG	HIV-1 integrase inhibitor;	[25]
		Antiinflammatory activity;	[68]
		Hepatoprotective activity;	[24]

	6-OHG	Hepatoprotective activity;	[46]

	8-OHD	Aldose reductase inhibitor;	[69]

	6-OHD	Suppression on adipogenesis of 3T3-L1 preadipocytes;	[70]
		Promoting differentiation of 3T3-L1 preadipocytes;	[71]
		Antitrypanosomal activity	[72]

	3′-OHD	Antitrypanosomal activity;	[72,73]
		Improving atopic dermatitis symptoms;	[74]

**Table 3.** Production of OHD and OHG by recombinant microorganisms harboring heterogeneous monooxygenase. Products that are underlined have the highest reported values.

Monooxygenase name	Monooxygenase sources	Recombinant hosts	Products	Yield (mg/L)	Ref.
CYP107H1 a	*B. subtilis*	*E. coli*	3′-OHD	<0.1	[75]
Nfa33880 a	*N. farcinica*	*E. coli*	8-OHD 6-OHD	0.76 0.56	[76]
CYP154 a	*N. farcinica*	*E. coli*	3′-OHD	0.99	[77]
CYP102D1 b	*S. avermitilis*	*E. coli*	8-OHD 6-OHD	2.42 1.18	[78]
CYP105D7 c	*S. avermitilis*	*S. avermitilis*	3′-OHD	9.3	[79]
CYP105D7 a	*S. avermitilis*	*S. avermitilis*	3′-OHD	37.5	[80]
CYP107Y1 a	*S. avermitilis*	*S. avermitilis*	3′-OHG	6.75	[21]
Sam5	*S. espanaensis*	*E. coli*	3′-OHD	75	[82]
CYP57B3 a	*A. oryzae*	*S. cerevisiae*	3′-OHG	N.D. d	[16]
CYP57B3 c	*A. oryzae*	*P. pastoris*	6-OHD	9.1	[87]

a Co-expressed with P450 reductase (camA/camB);

b Mutated CYP;

c Engineered fusion with P450 reductase;

d Not determined.
